# Pyrenoid loss impairs carbon-concentrating mechanism induction and alters primary metabolism in *Chlamydomonas reinhardtii*

**DOI:** 10.1093/jxb/erx121

**Published:** 2017-05-18

**Authors:** Madeline C Mitchell, Gergana Metodieva, Metodi V Metodiev, Howard Griffiths, Moritz T Meyer

**Affiliations:** 1 Department of Plant Sciences, University of Cambridge, Cambridge, UK; 2 School of Biological Sciences, University of Essex, Colchester, UK

**Keywords:** Carbon-concentrating mechanism, *Chlamydomonas*, photosynthesis, proteomics, pyrenoid, tandem mass spectrometry

## Abstract

Carbon-concentrating mechanisms (CCMs) enable efficient photosynthesis and growth in CO_2_-limiting environments, and in eukaryotic microalgae localisation of Rubisco to a microcompartment called the pyrenoid is key. In the model green alga *Chlamydomonas reinhardtii*, Rubisco preferentially relocalises to the pyrenoid during CCM induction and pyrenoid-less mutants lack a functioning CCM and grow very poorly at low CO_2_. The aim of this study was to investigate the CO_2_ response of pyrenoid-positive (*pyr+*) and pyrenoid-negative (*pyr–*) mutant strains to determine the effect of pyrenoid absence on CCM induction and gene expression. Shotgun proteomic analysis of low-CO_2_-adapted strains showed reduced accumulation of some CCM-related proteins, suggesting that *pyr–* has limited capacity to respond to low-CO_2_ conditions. Comparisons between gene transcription and protein expression revealed potential regulatory interactions, since Rubisco protein linker (EPYC1) protein did not accumulate in *pyr–* despite increased transcription, while elements of the LCIB/LCIC complex were also differentially expressed. Furthermore, *pyr−* showed altered abundance of a number of proteins involved in primary metabolism, perhaps due to the failure to adapt to low CO_2_. This work highlights two-way regulation between CCM induction and pyrenoid formation, and provides novel candidates for future studies of pyrenoid assembly and CCM function.

## Teaser

The chloroplast pyrenoid is essential for the accumulation of several low-CO_2_-inducible proteins as well as full carbon-concentrating mechanism (CCM) activity, suggesting two-way regulation between CCM and pyrenoid.

## Introduction

Upon exposure to low CO_2_, most unicellular eukaryotic algae are able to induce a carbon-concentrating mechanism (CCM), which generally includes a series of inorganic carbon transporters and carbonic anhydrases that deliver high concentrations of CO_2_ to the primary photosynthetic carboxylase Rubisco (Reinfelder, 2011). Rubisco itself is usually packaged in a chloroplast microcompartment known as a pyrenoid, which is thought to limit back-diffusion of CO_2_ (Badger *et al.*, 1998). CCMs are thus able to overcome Rubisco’s kinetic constraints and the slow diffusion of CO_2_ in water, allowing efficient photosynthesis and growth in CO_2_-limited environments (Giordano *et al.*, 2005).

Presently, 24 essential or putative components associated with the CCM of the eukaryotic model green alga *Chlamydomonas reinhardtii* (Dangard) have been identified and characterised (Wang *et al.*, 2015, 2016; Mackinder *et al.*, 2016). Nevertheless, there is a general consensus that to account for the multiple acclimation states to limiting CO_2_ and the ultrastructural remodelling that accompanies CCM induction—in particular with respect to pyrenoid formation and regulation of the CCM—many more CCM actors need to be identified. The earliest CCM components were identified through reverse genetics, by isolating mutants incapable of growing under air-level CO_2_ (Spalding *et al.*, 1983*a*, 1983*b*; Moroney *et al.*, 1986, 1989). Genome-wide high-throughput transcriptome studies (Miura *et al.*, 2004; Brueggeman *et al.*, 2012; Fang *et al.*, 2012) have since helped to identify novel low-CO_2_-responsive genes, some of which now form an integral part of the *Chlamydomonas* CCM model—for example, the chloroplast membrane transporter low-CO_2_-inducible A protein (LCIA) and the peri-pyrenoidal protein LCIB that may be involved in CO_2_ re/capture (Jin *et al.*, 2016). These studies also quantified the extent of genetic remodelling microalgae undergo when acclimating to low CO_2_ and provided key evidence that the *Chlamydomonas* CCM is under the high-level regulation of a nuclear factor, CIA5 (also known as CCM1). Studies that compare wild-type and high-CO_2_-requiring mutants under contrasting growth conditions (high CO_2_ for CCM-repressed and low CO_2_ for CCM-induced), coupled to high-throughput tools capable of resolving complex biological samples, therefore provide a powerful tool to advance our understanding of the algal CCM.

Of the three functional components of the algal CCM (inorganic carbon transporters, carbonic anhydrases, and a Rubisco micro-compartment), the pyrenoid is the least well characterised. Microscopy coupled to protein localisation has shown that CCM induction in *Chlamydomonas* coincides with the recruitment of almost all Rubisco to the pyrenoid, although around half of the cell’s Rubisco is retained in the pyrenoid even when the CCM is repressed at high CO_2_ or in the dark (Borkhsenious *et al.*, 1998; Mitchell *et al.*, 2014; Mackinder *et al.*, 2016). Mechanistic details of and factors needed for situating the pyrenoid within the chloroplast, for differential assembly of the seemingly ‘constitutive’ fraction and the ‘mobile’ fraction of the Rubisco matrix, or for anchoring the pyrenoid around modified photosynthetic lamellae are presently unknown, but some insight is progressively gained from mutants having some form of pyrenoid defect.

At least two chloroplast proteins are critical for pyrenoid formation: the Rubisco matrix linker EPYC1 (Mackinder *et al.*, 2016) and the Rubisco small subunit (Genkov *et al.*, 2010). Essential pyrenoid component 1 [EPYC1, originally identified as low-CO_2_-induced 5 or *LCI5* (Lavigne *et al.*, 2002)] was unambiguously implicated in pyrenoid formation when mass spectrometry (MS) analysis of purified wild-type pyrenoids found it to be a highly abundant component (Mackinder *et al.*, 2016). This intrinsically disordered protein is thought to either tether Rubisco holoenzymes into a para-crystalline arrangement or provide a scaffolding for the Rubisco enzymes to dock (Mackinder *et al.*, 2016). Mutants with little or no EPYC1 expression have a highly reduced pyrenoid, despite wild-type amounts of Rubisco per cell. In contrast, *Chlamydomonas* lines expressing higher-plant variants of the Rubisco small subunit (RBCS) have lost the capacity to form the Rubisco matrix that is distinctive of all algal pyrenoids, even though Rubisco levels are unaffected by the mutation (Genkov *et al.*, 2010). The high-CO_2_-requiring phenotype of EPYC1 and RBCS mutants provides direct evidence that a complete pyrenoid structure is essential for CCM function in *Chlamydomonas*. Indeed, pyrenoids probably enhance the whole-cell affinity for inorganic carbon in all species where present, as suggested by Morita *et al.* (1999) and Raven *et al.* (2012).

Pyrenoid defects have also been observed in mutants of regulatory proteins: the above-mentioned CCM master regulator CIA5/CCM1 (Fukuzawa *et al.*, 2001) and a putative methyltransferase CIA6 (Ma *et al.*, 2011), as well as several unmapped mutations that cause pyrenoid mis-localisation phenotypes (Yamano *et al.*, 2014). All these mutants also lack a fully functional CCM. Both *Ccm1* and *Cia6* mutants have reduced, deformed, or multiple pyrenoids, but some degree of Rubisco packaging is still generally visible. In contrast, mutations in the CCM critical carbonic anhydrase, CAH3, which is localised to lumen of trans-pyrenoidal lamellae, have seemingly no effect on pyrenoid phenotype (see Supplementary Fig. S1 at *JXB* online). Similarly, mutations in another thylakoid-associated protein, calcium-binding protein (CAS), reduce the affinity for inorganic carbon (Ci) but do not appear to affect pyrenoid formation (Wang *et al.*, 2016).

The higher-plant Rubisco small subunit substitution mutants are therefore presently the only *Chlamydomonas* lines where Rubisco packaging into a pyrenoid is totally lost, thereby representing a unique system to investigate the relationship between pyrenoid presence/absence and CCM induction and function. The aim of this study was thus to compare CCM gene expression and protein content for known CCM components in pyrenoid-positive (*pyr+*) and pyrenoid-negative (*pyr–*) *Chlamydomonas* lines. Using high-throughput MS analysis, we were then able to identify novel CCM candidates and gain insights into the cross-talk between the pyrenoid and normal metabolic processes in the cell. In addition to the structural function of the pyrenoid normally associated with the operation of a CCM, the combined findings from gene expression and proteome analysis allow us to suggest a regulatory cascade—incorporating Rubisco aggregation, full pyrenoid formation, and CCM expression—when cells adapt to low CO_2_ concentrations.

## Materials and methods

### Strains and culture conditions

The *pyr+* (wild-type) and *pyr–* (pyrenoid-less: *Spinacia oleracea*, *Arabidopsis thaliana*, and *Helianthus annuus* RBCS substitution) strains are near-isogenic and differ only in the gene encoding the Rubisco small subunit (Genkov *et al.*, 2010; Meyer *et al.*, 2012). Strains were maintained in the dark on Tris-acetate (Spreitzer and Mets, 1981) medium 1.5% (w/v) agar plates supplemented with Kropat’s trace elements (Kropat *et al.*, 2011). Experiments were performed on algae grown photoautotrophically in liquid cultures in an Innova 42 incubator (New Brunswick Scientific, Enfield, CT, USA) at 25 °C with 50–100 µmol photons m^−2^ s^−1^ illumination and shaking (125 rpm). Starter cultures were inoculated into Tris-minimal medium (Tris-acetate without the acetate) from freshly replated strains. Experimental cultures were inoculated from starter cultures and harvested at mid-log phase (approximately 1–2 × 10^6^ cells ml^−1^). Cultures were bubbled with air supplemented with 5% (v/v) CO_2_ (high-CO_2_-adapted) or bubbled with air [0.04% (v/v) CO_2_, low-CO_2_-adapted] for 3 h prior to harvest as this is sufficient for maximum CCM induction, including recruitment of Rubisco to the pyrenoid, and gene expression in wild-type cells (Mitchell *et al.*, 2014).

Growth curves were performed in a Multi-Cultivator MC 1000-OD connected to a Gas Mixing System GMS 150 (Photon Systems Instruments, Brno, Czech Republic). Test-tubes of Tris-minimal medium (60 ml) were inoculated from Tris-acetate starter cultures to an optical density (OD) of 0.05 at 680 nm and allowed to grow for 48 h to mid-log phase (0.6–1.0 OD_680nm_) at 25 °C. Cultures were then diluted to 0.05 OD_680nm_ and grown to mid-log phase a second time to ensure cells were acclimated to the new growth conditions. Cultures were bubbled with either 5% (v/v) CO_2_ or air and illuminated with 50 μmol photons m^−2^ s^−1^. Optical density measurements at 680 and 730 nm were recorded every 10 min. Doubling times were calculated from the slope of the curve of time versus log_2_OD for cells in the mid-log (linear) phase of growth.

### Immunoblots and qRT-PCR

Expression of CCM genes was determined using immunoblots (when antibodies were available) and quantitative reverse transcriptase polymerase chain reaction (qRT-PCR) as described in Mitchell *et al.* (2014). For detection of periplasmic CAH1 in wall-less *Chlamydomonas* strains, growth medium (10 ml) was collected after cells were pelleted and was concentrated using Amicon Ultra-15 centrifugal filter units (10 000 MWCO, EMD Millipore, Billerica, MA, USA). The growth medium was concentrated to equal volumes (approximately 500 μl) before loading aliquots (24 μl) for SDS-PAGE.

### Shotgun proteomic analysis using LC-MS/MS

Three biological replicates (individual cultures) of low-CO_2_-adapted *pyr+* and *pyr–* strains were harvested by centrifugation, snap-frozen in liquid nitrogen, and stored at −80 °C until analysis. Extraction and mass spectrometric identification and quantification of proteins, as well as statistical analyses, were carried out as described in McKew *et al.* (2013) and Metodieva *et al.* (2013). Briefly, proteins were extracted in a buffer containing SDS (2%, w/v), Tris-HCl (50 mM, pH 6.8), and protease inhibitors (Roche, Basel, Switzerland) and cell debris was removed by centrifugation. Proteins in the cleared supernatant were subjected to in-gel digestion with trypsin, then extracted, dried, and reconstituted in LC/MS-grade water containing 0.1% (v/v) formic acid. Peptides were separated on a 15-cm-long pooled-tip nanocolumn and analysed by electrospray ionization–tandem mass spectrometry on a hybrid high-resolution LTQ/Orbitrap Velos instrument (Thermo Scientific, Waltham MA, USA). Two injections (technical replicates) were performed for each sample. Proteins were identified using the UniProt database (www.uniprot.org/, last accessed 17 June 2014).

Protein abundance was quantified in two ways: label-free ion intensity and spectral counts. Label-free ion intensity (a measure of the number of ions of a given *m*/*z* detected during a particular time interval) is proportional to the absolute abundance of the protein, but the relationship is non-linear and this measure can only be used to compare the same ion species between different samples. The number of peptide-identifying spectra can be linearly correlated with the relative abundance of the protein in the sample but this measure is less sensitive to changes in proteins of low abundance. Analysis of both measures was included in order to capture differential expression of both high- and low-abundance proteins.

To identify proteins that were differentially expressed (*P*<0.05) between the *pyr+* wild-type and *pyr–* strains, a *t*-test was performed on the label-free intensity values, summed for the two technical replicates, for the three biological replicates of each strain. Imputation was used to avoid the use of zero values in the analysis but this did not significantly affect the results of the *t*-test (Karpievitch *et al.*, 2012). Secondly, a *G*-test (goodness-of-fit test) was performed on the spectral counts generated for each protein. This gave a different, but overlapping, list of differentially expressed proteins. Normalisation of spectral counts to a control protein—PSBO (oxygen-evolving enhancer protein of photosystem II, UniProt ID P12853) or GBLP (guanine nucleotide-binding protein subunit β-like protein, UniProt ID P25387; Schloss, 1990)—did not significantly affect the results.

Proteins of unknown function were analysed using computational tools to predict the following: general subcellular localisation (WoLF PSORT, Horton *et al.*, 2007); localisation to the mitochondria, chloroplast, or secretory pathway (green algae-specific PredAlgo, Tardif *et al.*, 2012); transmembrane domains (TMHMM v2.0, Krogh *et al.*, 2001); gene ontology (Phytozome, Goodstein *et al.*, 2012); structure (Argot2, Falda *et al.*, 2012); and function (Phyre^2^, Kelley and Sternberg, 2009).

## Results

**Fig. 1.**
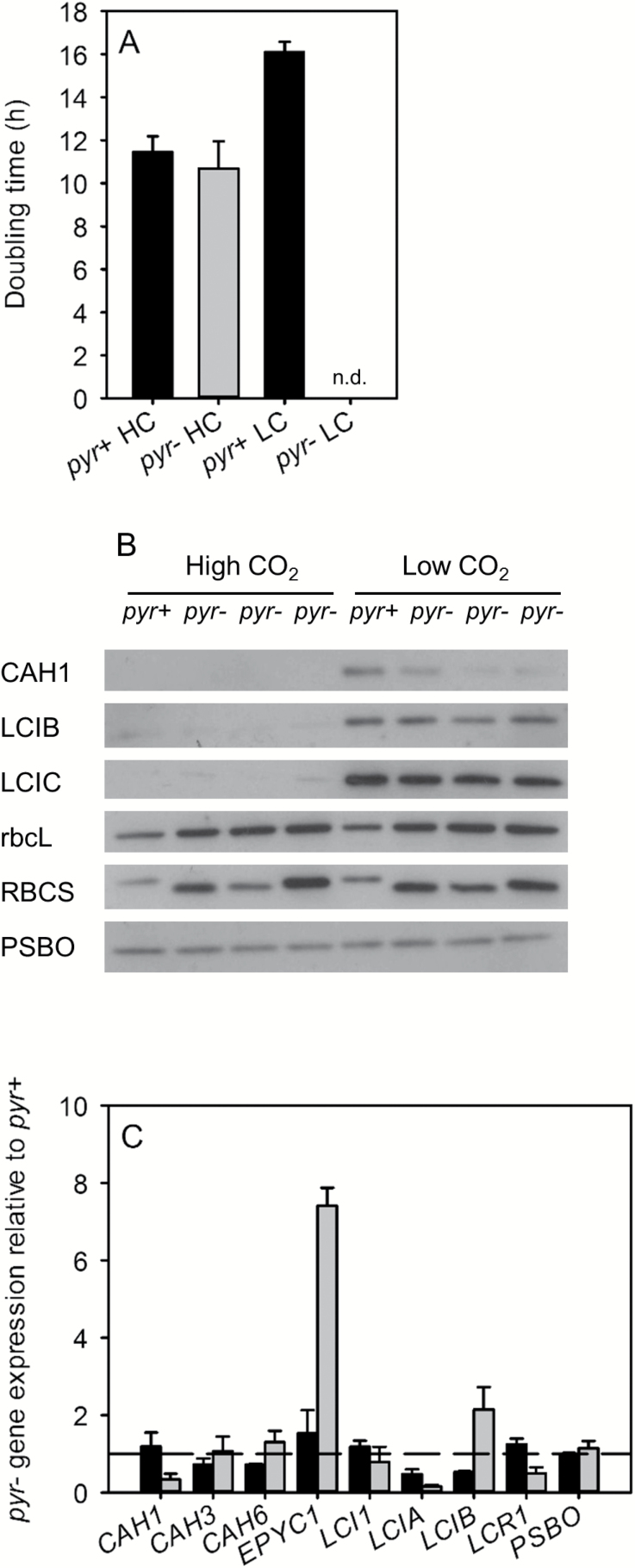
Growth and expression levels of CCM-related genes in pyrenoid-positive (*pyr+*) and three pyrenoid-negative (*pyr–*) strains adapted to high or low CO_2_. (A) Growth rates (doubling times) were measured for strains grown photoautotrophically in liquid cultures bubbled with high CO_2_ (HC) or low CO_2_ (LC). *Pyr–* cells had no detectable (n.d.) growth at low CO_2_. (B) Soluble proteins were separated using SDS-PAGE and probed with antibodies against Rubisco (rbcL and RBCS), CCM proteins (LCIB and LCIC), and a photosynthetic/control protein (PSBO). The abundance of the low-CO_2_-inducible periplasmic carbonic anhydrase, CAH1, was determined by probing concentrated growth medium. Pyrenoid-negative strains from left to right are: *Spinacia oleracea*, *Helianthus annuus*, and *Arabidopsis thaliana* RBCS substitution strains. (C) Relative mRNA abundance (mean ± s.e.) in high-CO_2_-adapted (black) and low-CO_2_-adapted (grey) cells was also determined using qRT-PCR.

Mutant *Chlamydomonas* strains with higher-plant RBCSs are unable to form pyrenoids and have a reduced photosynthetic affinity for Ci under CCM-induced conditions, leading to severely impaired growth (Meyer *et al.*, 2012; Fig. 1A). Preliminary characterisation of the CCM phenotype of these pyrenoid-less strains, at the molecular rather than whole-cell level, was performed using immunoblots and qRT-PCR to probe the expression of selected known CCM components (Fig. 1B, C). Under high CO_2_, the CCM proteins CAH1, LCIB, and LCIC were undetectable whereas Rubisco large and small subunits (rbcL and RBCS, respectively) as well as the control protein PSBO were present in *pyr+* and *pyr–* cells in roughly equal abundance. After 3 h exposure to low CO_2_, CAH1, LCIB, and LCIC had accumulated greatly in *pyr+* cells, but accumulation of CAH1 was much lower in *pyr–* strains. Accumulation of LCIC, but not LCIB, in low-CO_2_-acclimated *pyr–* lines was only slightly lower than *pyr+* cells. In contrast, the abundance of rbcL, RBCS, and PSBO showed no difference in relation to CO_2_ concentration.

qRT-PCR results generally correlated with immunoblot results, although there was variation in the transcriptional regulation of CCM-related genes in low-CO_2_-adapted *pyr–* strains (Fig. 1C). At low CO_2_, expression of *CAH1* mRNA was reduced more than 10-fold compared to *pyr+* levels, consistent with almost undetectable levels of CAH1 protein in the *pyr–* strains. *LCIA*, encoding an inorganic carbon transporter, was down-regulated in *pyr–* strains but *LCI1*, another Ci transporter, was not. *LCIB* mRNA levels were slightly elevated in *pyr–* strains compared to *pyr+* and there was no detectable difference in LCIB protein levels. The transcriptional regulator, *LCR1*, was slightly down-regulated in low-CO_2_-adapted *pyr–* cells while transcripts for the pyrenoid Rubisco linker component, *EPYC1*, were up-regulated in *pyr–* strains at low CO_2_. There was no significant difference in non-CO_2_-responsive carbonic anhydrase genes (*CAH3* and *CAH6*) and *PSBO* control gene expression between *pyr+* and *pyr–* strains.

### Overview of shotgun proteomic analysis

Twelve liquid chromatography-tandem mass spectrometry (LC-MS/MS) runs were performed on total proteins extracted from low-CO_2_-adapted *pyr+* and *pyr–* strains. This resulted in approximately 71 000 mass spectra confidently assigned to proteins present in *pyr+* (70 822 spectra) or *pyr–* (71 001 spectra) strains. Excluding *Chlamydomonas* and *Spinacia oleracea* RBCS, the spectra were distributed across 1376 predicted proteins (see Supplementary Dataset S1 for a complete list of the proteins identified). Spectral counts per run varied from 11 456 to 12 158.

**Fig. 2.**
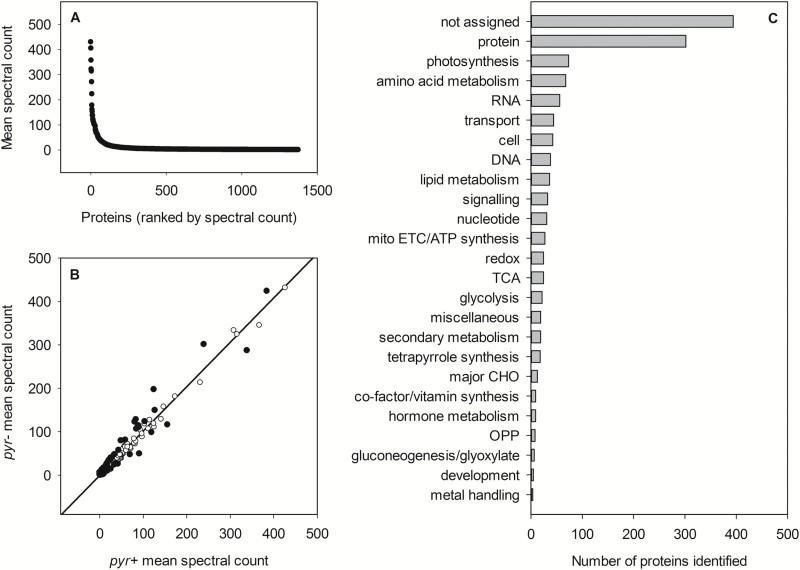
Overview of shotgun proteomic analysis of low-CO_2_-adapted pyrenoid-positive (*pyr+*) and pyrenoid-negative (*pyr–*) strains. (A) Rank abundance of total identified proteins. Mean spectral count is across two technical replicates of three *pyr+* and three *pyr–* replicate cultures. (B) Comparison of 1376 identified proteins (mean spectral counts) from *pyr+* and *pyr–* total proteomes. Differentially expressed proteins (*P*<0.05; closed circles) and non-differentially expressed proteins (open circles) were identified by a *G*-test. The equation for the line of best fit for the whole dataset is *y*=1.02*x*−1.25, *R*^2^=0.97. (C) Total proteins identified grouped according to function/pathway.

The mean spectral count (relative abundance) across all six samples was determined for each protein detected and showed the *Chlamydomonas* total protein samples to be dominated by a few highly abundant proteins (Fig. 2A). When ranked by mean spectral count and excluding RBCS, 56.4% of counts were associated with the top 50 proteins, 70.6% with the top 100 proteins, 94.1% with the top 500 proteins, and 5.9% with the remaining 884 proteins. Many of the most-abundant proteins (22 of the top 50) were associated with the photosystems/light-harvesting complexes. Ribosomal proteins also accounted for 100 of the top 500 proteins.

To investigate the difference between *pyr+* and *pyr–* samples, the mean spectral count for each protein detected in the *pyr–* strain was plotted against the mean spectral count for the same protein in *pyr+* cells (Fig. 2B). A line of best fit was plotted for all 1376 proteins and showed a strong linear relationship (*R*^2^=0.97, *y*=1.02*x*−1.25) between the relative abundance of proteins in the *pyr+* and *pyr–* samples. Only 84 proteins were detected in significantly different abundance based on spectral counts (*G*-test, *P*<0.05) in the two strains, while the remaining 1292 proteins were not differentially expressed. Based on label-free intensity values, 94 proteins were identified as differentially expressed (*t*-test, *P*<0.05), including 20 proteins already identified based on spectral counts, giving a total of 158 differentially expressed proteins, i.e. about one in ten. Of the differentially expressed proteins, only eight had a mean spectral count in *pyr+* greater than 100, 19 had mean spectral counts between 25 and 99, and the remaining 57 proteins had mean spectral counts of less than 25. Together with the relatively small number of differentially expressed proteins, this suggests that the proteomes of *pyr+* and *pyr–* strains differ only by a discrete number of significantly differentially expressed proteins after short-term adaptation to low CO_2_.

The total proteins detected in *pyr+* and *pyr–* strains were also assigned to functional categories using Mercator, an automated online tool for annotation of plant nucleotide and protein sequence data (Lohse *et al.*, 2014; Fig. 2C). Over a quarter (29%) of the protein sequences were not assigned to a functional group and the next largest group was protein assembly, modification, and degradation (22%). Proteins involved in RNA binding/transcription, DNA repair and chromatin structure, photosynthesis, transport, and primary metabolism (for example, amino acid, lipid, and nucleotide metabolism) were also moderately abundant, each accounting for between 2.2 and 5.3% of the total proteins identified. Secondary metabolism and other metabolic pathways accounted for most of the remaining proteins.

### Reduced accumulation of CCM proteins in the pyrenoid-less strain

**Fig. 3.**
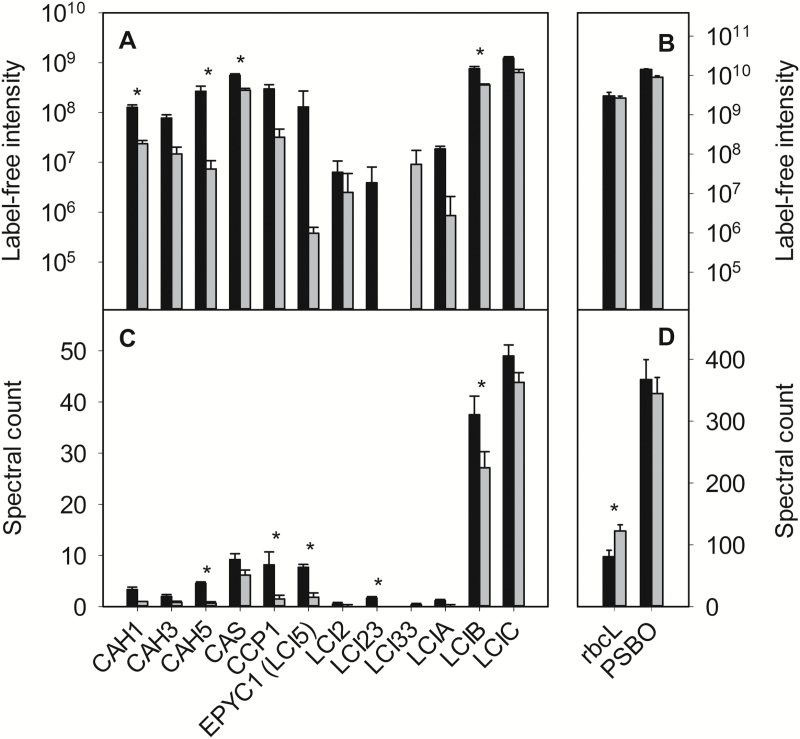
Relative quantification of 12 CCM proteins detected in low-CO_2_-adapted *pyr+* (black) and *pyr–* (grey) strains using LC-MS/MS. CCM and CO_2_-responsive proteins (A, C), non-CO_2_-responsive (rbcL), and photosynthetic/control (PSBO) proteins (B, D) are shown. Note the different scales of the *y*-axes. Two measures of protein abundance were used: label-free (ion) intensity, which is proportional to absolute abundance (A, B), and spectral count, which is a measure of the relative abundance in the sample (C, D). Values shown are the mean and standard error of six measurements (two technical replicates for three replicate cultures). Asterisks represent statistically significant differences (*P*<0.05) in protein abundance between the two strains according to a *t*-test on label-free intensity values or a *G*-test on spectral counts.

Nine proteins that are essential or highly likely to be required for the *Chlamydomonas* CCM (three carbonic anhydrases: CAH1, CAH3, CAH5; two inorganic carbon transporters: CCP1 and LCIA; and four pyrenoid-associated proteins: EPYC1, LCIB, LCIC, and CAS) as well as three low-CO_2_-induced proteins of unknown function (LCI2, LCI23, and LCI33) were detected using shotgun LC-MS/MS (Fig. 3A, C). Notably, the plasma membrane Ci transporters high-light-activated 3 (HLA3) and LCI1 as well as a second low-CO_2_-inducible mitochondrial Ci transporter (CCP2) were not detected. The chloroplast Ci transporter LCIA was present in only very low abundance (1–2 and 0–1 spectra per sample in *pyr+* and *pyr–*, respectively) while mitochondrial Ci transporter CCP1 was detected at slightly higher levels (4–15 and 0–3 spectra per sample in *pyr+* and *pyr–*, respectively). Using spectral counts as an estimation of the relative abundance of CCM proteins in both strains, LCIB and LCIC were much more abundant than any of the other CCM-related proteins (spectral counts for *pyr+* and *pyr–* strains ranged from 22–46 for LCIB and 38–54 for LCIC). The Rubisco large subunit (rbcL) was present in high abundance (spectral counts of 48–155) and was found to be significantly more abundant in the *pyr–* strain based on spectral counts, but not label-free intensity (Fig. 3B, D). The gene regulatory proteins CIA5/CCM1 and LCR1 were not detected in either strain. The control protein used for immunoblots, PSBO, was also detected in high abundance (approximately 350 spectral counts) and was not identified as differentially expressed.

In total, seven of the 12 detected CCM/LCI proteins (CAH1, CAH5, CAS, CCP1, EPYC1, LCI23, and LCIB) were significantly less abundant in low-CO_2_-adapted *pyr–* cells than in the *pyr+*. Except for LCI33, all the other CCM proteins detected appeared to be present in somewhat lower abundance in the *pyr–* strain, although the differences were not statistically significant.

### Changes to proteins involved in primary metabolism in the pyrenoid-less RBCS mutant strain

**Fig. 4.**
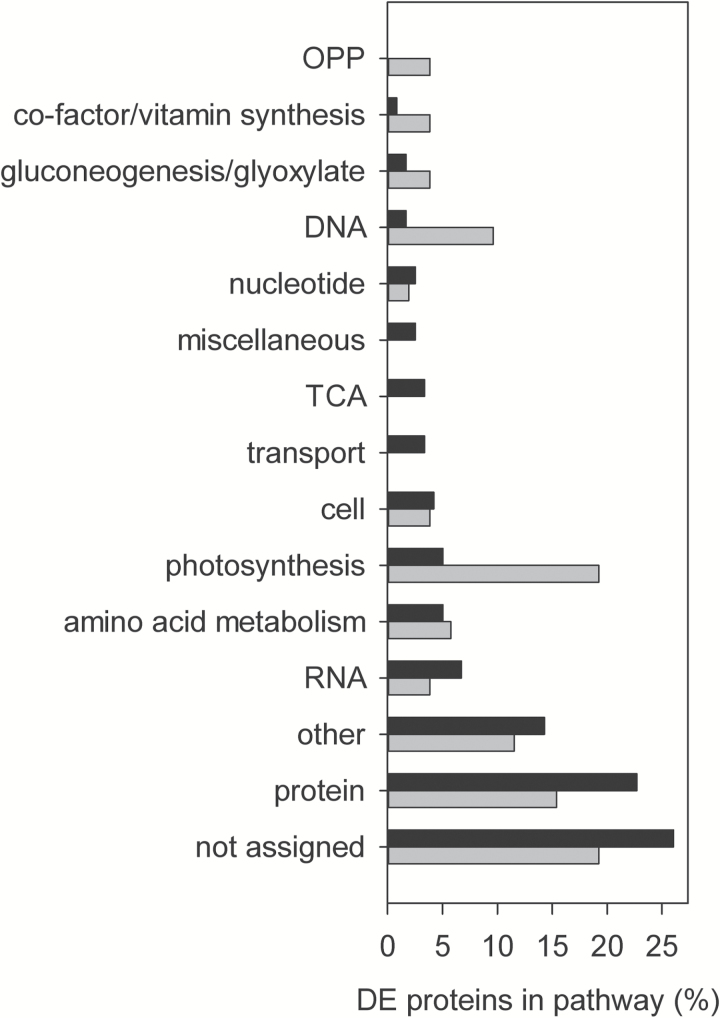
Functional classification of differentially expressed (DE) proteins in low-CO_2_-adapted *pyr+* (black) and *pyr–* (grey) cells.

Shotgun LC-MS/MS also identified 151 non-CCM proteins as differentially expressed between the *pyr+* and *pyr–* strains (see Supplementary Dataset S1). Classification of these proteins into pathways/functional groups revealed several differences in primary metabolism between low-CO_2_-adapted *pyr+* and *pyr–* strains (Fig. 4).

Fourteen proteins associated with the photosystems were differentially expressed between the two strains, but there was no overall systematic shift towards more reaction centres and/or antenna complexes in either *pyr+* or *pyr–* lines. Three proteins associated with photosystem I (PSI) were more abundant in *pyr+* (psaC, psaD, PSAN) and four were more abundant in *pyr–* cells (psaA, LHCI-3, LHCI-5, FNR1). Three proteins associated with photosystem II (PSII) were more abundant in *pyr+* (PSB28, PSBQ, PSBP) and four were more abundant in *pyr–* (REP27, psbD, LHCB4, psbC). Apocytochrome f (petA) was more abundant in *pyr–* and cytochrome b6f subunit V (PETO) was more abundant in *pyr+*. Two ATP synthase subunits (atpA, atpB) were more abundant in *pyr–*. Two fructose-bisphosphate aldolases (ALDCHL, A8JCY4) were more abundant in *pyr+* and may be involved in the Calvin–Benson–Bassham (CBB) cycle. Apart from the large subunit of Rubisco (rbcL), no other enzymes from the CBB cycle itself were identified as differentially expressed. However, the CBB regulatory protein, CP12, was found to be more abundant in *pyr–* cells.

Factors needed for transcription and protein synthesis (e.g. ribosomal proteins, elongation factors, RNA processing factors, and enzymes involved in protein folding) were much more abundant in the *pyr+* strain after 3 h of acclimation to low CO_2_. Other proteins involved in gene expression such as transcription factors and chromatin remodelling proteins were not generally found to be differentially expressed. The exception was one basal transcription factor (BTF3/A8JBX6), which was only detected in *pyr+* cells. This transcription factor appears highly conserved, with greater than 60% similarity to transcription factors from diverse plant species (for example, *Fragaria vesca*, *Zea mays*, and *Populus trichocarpa*), which is in contrast to the relatively unique CCM master regulator, CIA5, and CO_2_-responsive transcription factor, LCR1. CIA5 has homologues only in *Volvox carteri* (31.2% similarity) and *Coccomyxa subelleipsoidea* C-169 (8.6% similarity), while the closest homologues to LCR1 in *Chlamydomonas* and *V. carteri* are less than 10% similar. Unlike LCR1, BTF3 was not found to be CO_2_-responsive (Brueggeman *et al.*, 2012; Fang *et al.*, 2012) or to be co-expressed with other CCM genes during diurnal cycles (Zones *et al.*, 2015).

As with protein and RNA synthesis, the glycolysis and respiration pathways also appeared to be somewhat more highly represented in *pyr+* cells. Nine enzymes involved in glycolysis, the respiratory electron transport chain, and the tricarboxylic acid (TCA) cycle were more abundant in *pyr+*, while only four enzymes were more abundant in the *pyr–* strain. In contrast, proteins involved in the oxidative pentose phosphate, gluconeogenesis, and glyoxylate pathways were generally more abundant in *pyr–* cells, suggesting that photosynthesis may have been unable to provide the energy and carbon required for growth and that alternative energy sources were being used.

Other proteins of interest that were more abundant in the *pyr+* include: cytoskeletal proteins (α- and β-tubulin), a signalling protein (Ran-like small GTPase), and a regulator of gene expression (subunit C1 of the circadian RNA-binding protein CHLAMY 1). There was no difference in the number of nuclear-localised proteins (nucleosome and nucleolar proteins) differentially expressed in *pyr+* or *pyr–* cells (three each).

### Differentially expressed proteins of unknown function

Nearly one-quarter of the differentially expressed proteins identified in this study were predicted proteins of unknown function. Of these, 28 were more abundant in *pyr+* while only 10 were more abundant in *pyr–*. The relative abundance, localisation, homologies, and functional/structural annotation of each differentially expressed protein were analysed (see Supplementary Table S1).

Overall, most of the predicted proteins were present in low abundance in both *pyr+* and *pyr–* cells. The maximum mean spectral count was below 5 for 32 of the 38 proteins. The mean spectral counts for the remaining six proteins varied from 5.2 to 35.5. Up to 17 of the proteins of unknown function were predicted to be targeted to the chloroplast, eight to the cytosol, seven to the mitochondria, and five to the nucleus using two online prediction tools (Horton *et al.*, 2007; Tardif *et al.*, 2012). Only three proteins were predicted to have transmembrane domains using TMHMM v2.0 (Krogh *et al.*, 2001), which may implicate them in transport processes. The gene transcripts encoding 22 of these proteins are CO_2_-responsive (Brueggeman *et al.*, 2012; Fang *et al.*, 2012).

Computational predictions of protein structure (Phyre^2^; Kelley and Sternberg, 2009) yielded similar results to predictions of protein function (Argot2; Falda *et al.*, 2012) for 15 proteins and no or very low confidence results for a further five proteins. The remaining proteins either had conflicting or lower confidence predictions. Three proteins with predicted nuclear localisation were all more abundant in *pyr+* cells (A8HP50, A8IY40, and A8JEA7) and were identified as binding DNA or RNA. Other proteins that were preferentially accumulated in the *pyr+* strain have putative roles in amino acid synthesis (A8J3W1 and A8J3Y6), tRNA synthesis (A8IBN3), and proteolysis (A8JAW4, A8IGM2, A8IUN8).

Two proteins that may be involved in photosynthesis were also identified as more abundant in *pyr+* cells. One protein (A8HNG8) had a twin arginine motif in the predicted transit peptide, suggesting thylakoid localisation (Karlsson *et al.*, 1998), while functional predictions indicated association with the oxygen-evolving enhancer complex of PSII. The second protein (A8J995) is predicted to be a pentapeptide repeat protein with structural homology to a thylakoid lumenal protein from Arabidopsis as well as proteins from (CCM-positive) cyanobacteria *Cyanothece* and *Nostoc* species. Interestingly, one protein that was more abundant in *pyr–* (A8IQG4) had homology to an acetate transporter, although this was not a high-confidence prediction.

### Comparison with low-CO_2_-induced transcriptional changes

The 158 proteins identified as differentially expressed between the CCM-positive *pyr+* and CCM-negative *pyr–* strains were compared to gene transcripts that were differentially regulated in response to CO_2_ in a true wild-type (i.e. expressing both native RBCS isoforms) and inorganic carbon accumulation (*cia5*) mutant strains (Brueggeman *et al.*, 2012; Fang *et al.*, 2012; see Supplementary Dataset S2). Transcripts encoding 64 proteins identified as differentially expressed in this study had also been shown to be CO_2_-responsive by Brueggeman *et al.* (2012) (Supplementary Table S2), the majority of which are down-regulated in response to low CO_2_ (52 gene transcripts). In addition, transcripts encoding approximately one-quarter of the proteins (39) were identified as differentially expressed in a different wild-type strain by Fang *et al.* (2012) (Supplementary Dataset S2). The expression of 30 of these genes showed a significant response to CO_2_ concentrations (C-effect) and expression of 31 genes was affected by the *cia5* mutation (strain/S-effect; Supplementary Table S2). Overall, nine proteins that were more abundant in the wild-type were also induced at the transcript level in response to low CO_2_ in both transcriptome datasets. This included the CCM/low-CO_2_-induced proteins CAH1, CAH5, CCP1, EPYC1, LCI23, and LCIB. The other proteins found in all three datasets were malate dehydrogenase (MDH2; A8ICG), β-ureidopropionase (A8HPY4), and a predicted protein (A8IGV4). All three proteins were present in relatively low abundance (mean spectral counts of 1.0–1.3 in the wild-type and 0.0 in *pyr–*). Malate dehydrogenase is involved in the TCA cycle, although this was the least-abundant isozyme detected, suggesting that it may have an alternative role. β-ureidopropionase is involved in uracil degradation and the predicted protein (A8IGV4) has sequence similarity to an α-glucan water dikinase/pyruvate phosphate dikinase (Supplementary Table S1). To test whether the current set of differentially expressed proteins was also differentially expressed at the transcript level in response to the same change in CO_2_ regime, it was compared to a randomly selected set of non-differentially expressed proteins, but no difference was found (Supplementary Table S2).

## Discussion

**Fig. 5.**
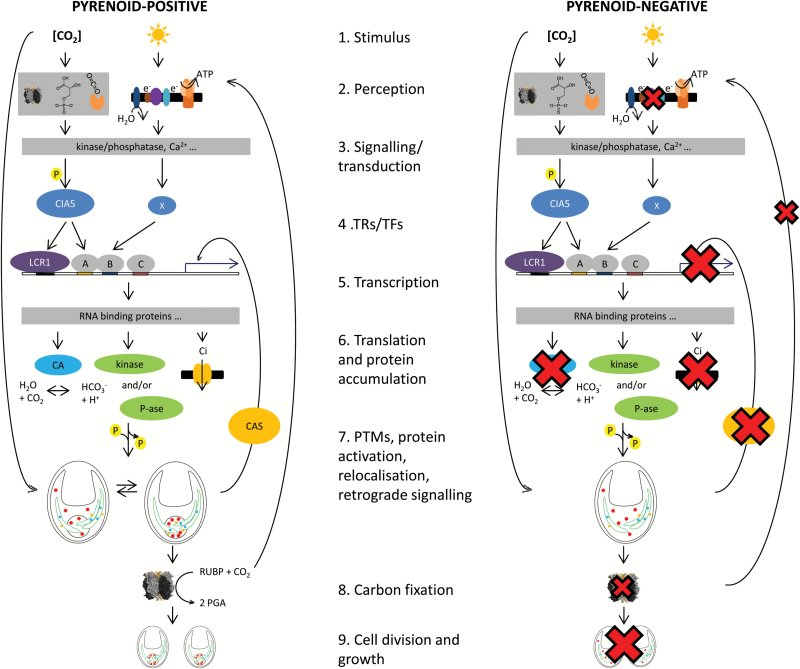
Summary of low-CO_2_-induced CCM induction in pyrenoid-positive and pyrenoid-negative strains. Pyrenoid-positive (*pyr+*) cells induce a CCM at low CO_2_ in the light (1), which may be a response to low external or internal CO_2_ concentrations, sensed via reduced CO_2_ assimilation, altered metabolite pools, or a receptor (2). Signal transduction may occur via de/phosphorylation and Ca^2+^/calmodulin (3) and results in the activation of the CCM master regulator (CIA5/CCM1) and other as yet unknown transcription regulators/factors (4). Major transcriptional changes occur in response to low CO_2_, including the up-regulation of carbonic anhydrase and Ci transporter genes (5). The accumulation of these proteins (6) as well as their correct post-translational modification (PTM) and re/localisation (7) allows high rates of carbon fixation to occur (8) and cells to continue to grow and divide normally (9). In contrast, low-CO_2_-inducible gene transcripts and proteins do not accumulate to normal levels in pyrenoid-negative (*pyr–*) cells, suggesting that they are impaired in one or more of these steps. Large crosses indicate a phenotype described in this paper while small crosses indicate a phenotype described previously. The absence of a pyrenoid could impair carbon fixation at low CO_2_ and decrease electron transport rates (Caspari *et al.*, 2017). Lower electron transport rates as well as reduced CAS abundance (this study) could lead to reduced accumulation of CCM gene transcripts (this study). CCM proteins may then fail to accumulate to wild-type levels (this study) and may also fail to correctly localise in the absence of a pyrenoid (to be determined in future work). Pyrenoid-negative cells are unable to maintain sufficiently high rates of photosynthesis (Genkov *et al.*, 2010) and growth is limited (this study; Meyer *et al.*, 2012). Elements of this model for which there is currently limited evidence are shaded grey.

The *pyr+* and *pyr–* strains are near-isogenic yet exhibit large differences in phenotype when grown at low CO_2_, which this study has shown is associated with a relatively small number of distinct changes to the proteome. Twelve CCM-related or low-CO_2_-induced proteins were detected in this study, of which seven failed to accumulate to *pyr+* levels in the *pyr–* mutant. This impaired induction of some CCM proteins could also contribute to the observed reduction in CCM activity. However, CCM proteins were only reduced in abundance, so the effect on overall CCM activity is likely to be small compared to the complete absence of Rubisco aggregation in the pyrenoid matrix. The current understanding of CCM induction in *pyr+* cells and steps that may be compromised in *pyr–* cells are summarised in Fig. 5 and discussed below. We propose that the key findings relate to the inability of *pyr–* cells to aggregate Rubisco into a pyrenoid, leading to subsequent interactions between gene expression and protein accumulation that help to define regulatory processes leading to CCM induction and expression.

In *pyr+ Chlamydomonas*, full CCM induction occurs in response to low CO_2_ in the light (Fig. 5.1). In *pyr–* cells, it appears that the lack of Rubisco aggregation in the pyrenoid has downstream effects on the accumulation of CCM gene transcripts and protein accumulation. When CO_2_ concentration drops, both *pyr+* and *pyr–* strains may initially respond by inducing CCM-related genes. In *pyr+*, this increases affinity for Ci and allows cells to continue to photosynthesise. In contrast, *pyr–* cells may be unable to maintain normal carbon fixation rates without pyrenoid-facilitated CCM activity. This might lead to increased non-photochemical quenching and reduced electron transport rates (see Caspari *et al.*, 2017), which reflects the extent of CCM induction and internal CO_2_ availability (Fig. 5.3). This is consistent with the observation that while electron transport appears necessary for carbon accumulation (Spalding and Ogren, 1982; Spalding *et al.*, 1984), probably due to the demand for high-energy compounds to energise Ci transport, electron transport may also be required for expression of CCM components (Dionisio-Sese *et al.*, 1990; Eriksson *et al.*, 1998; Im and Grossman, 2002).

The requirement of photosynthesis for full CCM expression might explain the similarities between the CCM phenotype of the *pyr–* RBCS substitution mutant and that of other Rubisco mutants. In mutants that have reduced Rubisco levels or that produce only an inactive enzyme, several low-CO_2_-inducible proteins also fail to accumulate (Chen *et al.*, 1990; Tavío *et al.*, 1996; Villarejo *et al.*, 1996). Reduced expression of CCM genes, unrelated to the primary lesion or mutation, has also been observed in other CCM mutants (Spalding *et al.*, 1991; Miura *et al.*, 2004; Ma *et al.*, 2011), although the mechanisms have not yet been identified and this phenomenon remains largely unexplored.

The *pyr–* phenotype also resembles a regulatory or signalling mutant (Fig. 5.4–7). In wild-type cells, Ca^2+^ accumulation in the pyrenoid is important for CAS-facilitated retrograde signalling to maintain mRNA levels of key Ci transporters (*HLA3* and *LCIA*) during CCM induction (Wang *et al.*, 2016). The observed reduced expression of CAS in the *pyr–* strain could impair CCM induction in these cells (Fig. 5.7) and perturbations in chloroplast/pyrenoid Ca^2+^ concentrations may have additional effects on signal transduction and regulation of photosynthesis (Kim *et al.*, 2010; Hochmal *et al.*, 2015).

A key finding in support of this hypothesis is the differential expression of gene transcripts for two proteins that normally relocalise to the pyrenoid during CCM induction, EPYC1 and LCIB. Both accumulated to lower protein levels in *pyr–* (Fig. 3A, 3C; see Fig. 5.6, 5.7), with *EPYC1* but not *LCIB* up-regulated at the transcriptional level (Fig. 1C), suggesting distinct modes of regulation. Based on transcriptomic analysis, Fang *et al.* (2012) assigned these proteins to separate CCM clusters: *EPYC1* belonged to a primarily metabolic group of genes and *LCIB* to a primarily signalling group. Over-accumulation of *EPYC1* mRNA in *pyr–* perhaps compensates for the inability of the protein to interact with and aggregate Rubisco within the pyrenoid (Mackinder *et al.*, 2016), either because of the results of arrested translation and/or rapid degradation of EPYC1. It will be important to investigate whether the residual EPYC1 present in *pyr–* is in an active or inactive form.

This observation provides insights into the hierarchical arrangement of factors needed for normal pyrenoid biogenesis. The *pyr–* mutants retain, for example, the network of thylakoid tubules at the base of the chloroplast and preferential deposition of large starch granules in the area where a pyrenoid would normally form (Genkov *et al.*, 2010; Meyer *et al.*, 2012; Caspari *et al.*, 2017). The lower expression of LCIB relative to LCIC is also consistent with the requirement for feedback from correct Rubisco aggregation for complete assembly of the LCIB/LCIC complex. LCIB has been proposed to act as a β-carbonic anhydrase and, in complex with LCIC, may contribute to a CO_2_ recapture system surrounding the pyrenoid (Wang and Spalding, 2014; Jin *et al.*, 2016).

In addition to several CCM proteins, LC-MS/MS analysis identified a further 151 differentially expressed proteins that accounted for approximately 37% of the total spectral counts and reflected the altered expression of a number of biological pathways that were not directly related to the CCM. Similar to the reduced accumulation of CCM proteins, enzymes involved in primary metabolism also tended to be present in lower abundance in *pyr–* cells. In interpreting these results, the metabolism of the *pyr–* strain is assumed to be equivalent to that of the *pyr+* strain at high CO_2_ based on equivalent growth rates, gene expression, chlorophyll fluorescence, and electron transport rates under conditions when the CCM, and presumably the pyrenoid, are not required (Fig. 1; Caspari *et al.*, 2017).

Low-CO_2_-adapted *pyr+* cells appear to be more metabolically active overall with a greater number of proteins up-regulated in the TCA cycle, respiration, fatty acid, amino acid, and protein synthesis pathways. This may be due to higher rates of carbon fixation in *pyr+* cells but it could also be due to the need for increased protein synthesis during CCM induction (Fig. 5.6, 5.8). Greater metabolic activity in *pyr+* cells would also be consistent with the strongly impaired growth of *pyr–* at low CO_2_ (Meyer *et al.*, 2012). If *pyr–* cells are largely unable to adapt to low CO_2_ and divide only slowly, a general reduction in the abundance of primary metabolic enzymes after 3 h at low CO_2_ might be indicative of the reduced growth of this strain (Fig. 5.9).

The reduced CCM induction at the protein level identified in this study indicates that the RBCS substitution mutants could be used to identify new components of the CCM. Novel CCM candidates are likely to be those proteins that are present in greater abundance in low-CO_2_-adapted *pyr+* cells. Proteins encoded by genes previously identified as low-CO_2_-induced (LCI23 and LCI33) are obvious candidates. Expression of *LCI23*, encoding a septin-like protein, was strongly induced by low CO_2_ in the wild-type but not the *cia5* mutant strain and annotation suggests it encodes a transmembrane protein (Fang *et al.*, 2012). In addition, the 28 proteins of unknown function that are more abundant in *pyr+* may also include novel CCM components. Future comparison of these proteins with the sequences of 1000 plant transcriptomes (1KP project) may give additional insight into which ones are most likely to be CCM-specific.

While LC-MS/MS is a very sensitive technique, the dynamic range is reduced for complex mixtures of proteins, especially those that are dominated by a few highly abundant proteins (Schulze and Usadel, 2010). These limits to detection distinguish proteomics approaches from microarray and RNA sequencing experiments, which are able to detect up to 16 000 individual gene transcripts in *Chlamydomonas* (Miura *et al.*, 2004; Brueggeman *et al.*, 2012; Fang *et al.*, 2012). However, the lower coverage of a proteomics experiment can be overcome by cellular fractionation and enrichment prior to mass spectrometric analysis, and such large-scale proteomics approaches will be required in the future to complement the substantial microarray and transcriptome datasets that have already been generated (Miura *et al.*, 2004; Yamano *et al.*, 2008; Brueggeman *et al.*, 2012; Fang *et al.*, 2012; Zones *et al.*, 2015). Better quantification of protein abundance should enable a distinction to be made between what is primarily a transcriptional response and what is occurring at the functional (protein) level. This may narrow down the number of CCM candidates further than the current list of >1000 published genes that are transcriptionally up-regulated in response to low CO_2_ (Miura *et al.*, 2004; Wang *et al.*, 2005; Yamano *et al.*, 2008; Yamano and Fukuzawa, 2009; Brueggeman *et al.*, 2012; Fang *et al.*, 2012). Indeed, the results of this LC-MS/MS experiment demonstrate that a genome-scale proteomics approach can complement existing studies, as well as build on current knowledge and understanding of CCM regulation.

## Conclusions

Analysis of the proteomes of low CO_2_-adapted *pyr+* and *pyr–* strains has provided further insights into CCM and metabolic regulation in a pyrenoid-less mutant, as well as demonstrating that a shotgun LC-MS/MS approach can be effectively adapted to study low-CO_2_ acclimation in *Chlamydomonas*. Despite differing from *pyr+* in the sequence of only a single gene, the *pyr–* mutant shows large differences in cellular ultrastructure (loss of pyrenoid) and gene expression. The *pyr–* mutant fails to fully induce CCM components in response to low CO_2_ and this, along with the complete lack of Rubisco aggregation, is likely to be responsible for the absence of any detectable increase in photosynthetic affinity for Ci in low-CO_2_-adapted cells. The apparent down-regulation of primary metabolism also observed in the CCM-negative *pyr–* mutant may simply be a downstream result of this impaired carbon fixation, but further evidence is needed. Analysis of post-translational modification of CCM components could build on this study and may uncover additional factors determining the final localisation, activity, and abundance of CCM proteins.

## Supplementary data

Supplementary data are available at *JXB* online.

Table S1. Structural, localisation, and functional predictions for differentially expressed proteins of unknown function.

Table S2. Summary of comparison of proteins identified in *pyr+* versus *pyr–* by LC-MS/MS with genes differentially expressed in wild-type and *cia5* mutant strains in response to low CO_2_.

Fig. S1. Transmission electron micrograph of CAH3-deletion mutant.

Dataset S1. List of all proteins identified by LC-MS/MS proteomic analysis of *pyr+* and *pyr–* strains.

Dataset S2. Comparison of low-CO_2_-adapted *pyr+* and *pyr–* LC-MS/MS proteomic data with transcriptomic datasets.

## Author contributions

MCM, HG, and MTM conceived of the study and designed the experiments. MCM grew and harvested the strains, performed immunoblots, functional proteomic data analysis, and wrote the manuscript. MTM performed the qRT-PCR. GM performed the protein extraction and proteomic analysis. MVM performed the statistical and initial data analysis. All authors read and approved the manuscript.

## Supplementary Material

Supplementary_tables_S1_S2_Figure_S1

supplementary_dataset_S1_S2

## Abbreviations:

CAH: carbonic anhydrase
CBB cycle: Calvin–Benson–Bassham cycle
CCM: carbon (dioxide)-concentrating mechanism
CCP: chloroplast carrier protein
Ci: inorganic carbon
CIA: inorganic carbon accumulation mutant
EPYC: essential pyrenoid component
HLA: high-light-activated
LC-MS/MS: liquid chromatography-tandem mass spectrometry
LCI: low-CO_2_-inducible
PS: photosystem
qRT-PCR: quantitative reverse transcriptase polymerase chain reaction
rbcL: Rubisco large subunit
RBCS: Rubisco small subunit
TCA cycle: tricarboxylic acid cycle
Tris: 2-amino-2-hydroxymethyl-propane-1,3-diol.
