# Supplementary material for: Pyrenoid loss impairs carbon-concentrating mechanism induction and alters primary metabolism in Chlamydomonas reinhardtii
Source: J Exp Bot. 2017 May 18;68(14):3891–902. doi: 10.1093/jxb/erx121 (PMC5853466; doi:10.1093/jxb/erx121)
Supplement: Supplementary_tables_S1_S2_Figure_S1 [file erx121_suppl_supplementary_tables_s1_s2_figure_s1.pdf]

Journal of Experimental Botany

Pyrenoid loss impairs carbon-concentrating mechanism induction and alters primary metabolism in *Chlamydomonas reinhardtii*

Madeline C. Mitchell, Gergana Metodieva, Metodi V. Metodiev, Howard Griffiths, Moritz T. Meyer

Supplementary Table S1. Predicted localisation, function and structure of the 38 predicted proteins identified as more abundant in low CO_2-adapted pyrenoid-positive (*pyr+*) or pyrenoid-negative (*pyr-*) cells. Mean spectral counts (S.C.) for each strain are also given. Localisation was performed using WoLF PSORT (Horton et al., 2007) and PredAlgo (Tardif et al., 2012) and predicted chloroplast-localised proteins were manually checked for dual partite thylakoid signal peptides (thyl) as per Karlsson et al. (1998). Other predicted localisations were abbreviated as follows: chlo, chloroplast; cyto, cytosol/cytoplasm; ER, endoplasmic reticulum; extr, extracellular; mito, mitochondrion; nucl, nucleus; plas, plasma membrane; secr, secretory pathway; vacu, vacuole. Localisations that are different between the two tools are italicised. Transmembrane domains (TMD) were predicted using TMHMM v2.0 (Krogh et al., 2001). Phytozome function was determined using the PANTHER and GO annotations available for the latest genome (v5.5). Argot[2] (Falda et al., 2012) was used to predict protein function and results with a score ≥200 are listed from highest to lowest (annotations in parentheses indicate a low score or no hit found). Phyre[2] (Kelley and Sternberg, 2009) was used to predict protein structure and the top hits (high % confidence, high % coverage) are listed in each case. Proteins with transcripts identified as CO_2-responsive are listed under the heading 'CO_2 DE': B, Brueggeman et al., (2012);F, Fang et al., (2012); U, upregulated in response to low CO_2; D, downregulated in response to low CO_2. Proteins with consistent annotations across the three prediction programs are shaded in grey. Protein are listed alphabetically by UniProt ID.

UniProt ID	Up in *pyr+*/ *pyr-*	*pyr+* S.C.	*pyr-* S.C.	WoLF PSORT Local.	Pred Algo Local.	TMD	Phytozome function (PANTHER; KEGGORTH/KEG/GO)	Argot2 (molecular function; biological process; cellular component)	Phyre[2] structure (%confidence; %coverage)	CO_2 DE
A8HMM7	*pyr-*	0	0.8	chlo	chlo	1	no functional annotation	(not found); (not found); (not found)	no high confidence hits (<9%)	B (D)
A8HNG8	*pyr+*	5.2	1.7	chlo (thyl)	chlo	1	oxygen evolving enhancer protein 3 (PSBQ) (uncharacterised thylakoid lumenal polypeptide)	calcium ion binding; photosynthesis; photosystem II oxygen evolving complex	no high confidence hits (<12%)	B (D) F(D)
A8HP50	*pyr+*	0.5	0	nucl	other	0	RNA-binding protein LUC7-related; spliceosome subunit	mRNA binding; mRNA splice site selection; U1 snRNP	rubrerythrin (88%; 28%), C2H2, DNA/RNA binding (82%; 8%)	B (D) F(D)
A8HSJ6	*pyr+*	0.8	0	extr	secr	0	dolichyl-phosphate-mannose-protein mannosyltransferase, stromal cell-derived factor 2-related; membrane	mannosyltransferase/calcium channel; PAMP/defense response to fungus or bacterium; membrane/ER	stromal cell-derived factor 2-like protein (100%; 82%)	B (D)
A8I0A3	*pyr+*	0.8	0	cyto	other	0	sialic acid synthase-related; phospholipid:diacylglycerol acyltransferase	acetyl transferase; metabolic process; cytoplasm	Galactoside acetyltransferase-like (100%; 95%), one TMD spanning PM	-

UniProt ID	Up in *pyr+*/ *pyr-*	*pyr+* S.C.	*pyr-* S.C.	WoLF PSORT Local.	Pred Algo Local.	TMD	Phytozome function (PANTHER; KEGGORTH/KEG/GO)	Argot2 (molecular function; biological process; cellular component)	Phyre[2] structure (%confidence; %coverage)	CO_2 DE
A8I3W8	*pyr+*	0.7	0	chlo/ extr	secr	0	no functional annotation	(not found); (not found); (not found)	no high confidence hits (<41%)	F (U)
A8I9T2	*pyr-*	0	0.5	*mito*	*chlo*	0	Kinesin motor protein, kinesin-9 (Kif9) family	microtubule motor/ATP binding; microtubule-based movement; kinesin complex/microtubule/cytoplasm	chimera of maltose-binding periplasmic protein and kinesin (100%; 40%), P-loop containing nucleoside triphosphate hydrolases	-
A8IBN3	*pyr+*	5.2	3.3	cyto	other	0	prolyl tRNA synthetase	proline -tRNA ligase/ amino acyl-tRNA ligase; tRNA aminoacylation/ translation; cytoplasm	bifunctional glutamate/proline-tRNA ligase (100%; 98%)	F (D)
A8ICU7	*pyr-*	0	0.8	*mito*	*other*	0	serine protease	serine-type peptidase; proteolysis; thylakoid lumen/mitrochondrion	photosystem II d1 protease (100%; 97%)	B (D)
A8IGE2	*pyr+*	1.2	0	ER/ plas	other	0	survival protein surE, 5'-nucleotidase	hydrolase; dephosphorylation/metabolic process; cytoplasm	5'-nucleotidase surE (100%; 79%)	B (D) F(D)
A8IGM2	*pyr+*	1.5	0	chlo	chlo	0	aspartyl aminopeptidase, zinc metalloprotease	zinc ion binding/hydrolase/ aminopeptidase; proteolysis; cystosol/vacuolar membrane/ vacuole	aspartyl aminopeptidase (100%; 80%)	-
A8IGV4	*pyr+*	1	0	cyto	other	0	phosphoenolpyruvate dikinase-related	kinase/ATP binding/catalytic; phosphorylation/starch catabolic process; chloroplast envelope	pyruvate phosphate dikinase, N-terminal domain (100%; 82%)	B (U) F (U)

UniProt ID	Up in *pyr+*/ *pyr-*	*pyr+* S.C.	*pyr-* S.C.	WoLF PSORT Local.	Pred Algo Local.	TMD	Phytozome function (PANTHER; KEGGORTH/KEG/GO)	Argot2 (molecular function; biological process; cellular component)	Phyre[2] structure (%confidence; %coverage)	CO_2 DE
A8IHW6	*pyr+*	1	0	nucl/ plas	other	0	no functional annotation	(ATP binding); (vesicle-mediated transport); mitochondrion	no high confidence hits (<47%)	B (D)
A8IQG4	*pyr-*	22.3	35.5	cyto	other	6	GPR1/FUN34/yaaH family, membrane, mannosyl-3-phosphoglycerate phosphatase	(oxidoreductase); (plasma membrane acetate transport); membrane	no high confidence hits (<33%)	-
A8IRW1	*pyr+*	1.2	0	vacu	other	0	no functional annotation	(catalytic activity); (metbolic process); (not found)	DNA repair protein Rad51, N-terminal domain (94%; 26%)	-
A8ITZ2	*pyr+*	0.7	0	*chlo*	*other*	0	no functional annotation	Ran GTPase activator; positive regulation of Ran GTPase activity; (kinetochore; cytoplasm)	No high confidence hits (<40%)	-
A8IUN8	*pyr+*	0.5	0	chlo/ cyto	other	0	26s proteasome non-ATPase regulatory subunit	(not found); multicellular organismal development; proteasome complex/ nucleus/cytoplasm	26s proteasome regulatory subunit rpn5 (100%; 83%)	-
A8IV51	*pyr+*	0.7	0	*mito*	*secr*	0	prolyl 4-hyrdoxylase alpha subunit; oxidoreductase activity	itron ion binding/oxidoreductase; oxidation-reduction process; endosome/Golgi	prolyl-4 hydroxylase (100%; 73%)	F (D)
A8IWP6	*pyr-*	0	1.3	*nucl*	*mito*	0	calcium independent phospholipase A2 (IPLA2)-related	(not found); lipid metabolic process; (not found)	VipD (phospholipase) (100%; 50%)	F (U)

**UniProt ID**	**Up in *pyr+/ pyr-***	***pyr+* S.C.**	***pyr-* S.C.**	**WoLF PSORT Local.**	**Pred Algo Local.**	**TMD**	**Phytozome function (PANTHER; KEGGORTH/KEG/GO)**	**Argot2 (molecular function; biological process; cellular component)**	**Phyre[2] structure (%confidence; %coverage)**	**CO_2 DE**
A8IY40	*pyr+*	0.7	0	chlo/ cyto	other	0	RNA recognition motif	nucleic adic binding; (not found); nucleus	RNA binding protein (100%; 95%), splicing factor (100%; 94%), ribonucleoprotein (100%; 91%)	B (D) F(D)
A8J3W1	*pyr+*	1.5	0	cyto	other	0	asparagine synthetase	(transferase activity); asparagine biosynthetic process/cellular amino acid biosynthetic process; cytosol/nucleus	asparagine synthetase b (100%; 86%)	-
A8J3Y6	*pyr+*	6.7	1.8	*mito*	*chlo*	0	AIR synthase domain; Phosphoribosylformylglycinamidine synthase	phosphoribosylformylglycinamidine synthase/catalytic; 'de novo' IMP biosynthetic process; cytoplasm/chloroplast/ mitochondrion	phosphoribosylformylglycinamidine synthase (100%; 89%)	-
A8J462	*pyr-*	0	1	*chlo*	*other*	0	alanyl tRNA synthetase	zinc ion binding/alanine-tRNA ligase; tRNA aminoacylation/translation; cytoplasm/chloroplast/ mitochondrion	alanyl-tRNA synthetase (100%; 70%)	B (D)
A8J4M0	*pyr+*	1.8	0.2	nucl	other	0	no functional annotation	(signal transducer); (signal transduction); (membrane)	keratin, type II cytoskeletal 5 (94%; 47%)	B (D)
A8J5B8	*pyr-*	20.3	32.2	*cyto*	*mito*	0	AMP-activated protein kinase, gamma subunit	adenyl nucleotide binding; (metabolic process); (membrane/cytoplasm)	nuclear protein snf4 (100%; 72%), AMP binding, CBS domain pair	B (D) F(D)

UniProt ID	Up in *pyr+*/ *pyr-*	*pyr+* S.C.	*pyr-* S.C.	WoLF PSORT Local.	Pred Algo Local.	TMD	Phytozome function (PANTHER; KEGGORTH/KEG/GO)	Argot2 (molecular function; biological process; cellular component)	Phyre[2] structure (%confidence; %coverage)	CO_2 DE
A8J6M8	*pyr+*	0.5	0	vacu	other	0	N-linked oligosaccharide processing/ glucosidase II beta subunit-like protein	(metal ion binding); N-glycan processing/defense response to bacterium; ER	low-density lipoprotein receptor-related protein (100%; 80%)	B (D)
A8J7P4	*pyr+*	2	1.2	cyto	other	0	no functional annotation	(ATP binding); (ATP catabolic process); (cytoplasm)	no high confidence hits (<37%)	B (D) F(D)
A8J7X3	*pyr-*	0	1.3	plas	secr	0	n/a	metal ion binding/(hydrolase); (signal transduction); (membrane)	ferredoxin reductase FAD-binding domain-like (95%, 4%)	F (U)
A8J995	*pyr+*	0.7	0	chlo	chlo	0	pentapeptide repeats (8 copies)	(not found); (not found); (chloroplast thylakoid lumen/membrane)	secreted effector protein (100%; 64%), A.t. pentapeptide repeat thylakoid lumenal protein (100%; 54%), cyanobacterial homologues (Cyanothece and Nostoc sp.)	-
A8JAW4	*pyr+*	14	10	cyto	other	0	protease family M24, proliferation-associated protein 2G24; metallopeptidase	metalloexopeptidase/aminopeptidase; proteolysis; nucleolus/plasma membrane	putative curved dna-binding protein (100%; 89%), ribosome (100%; 91%), aminopeptidase (100%; 87%)	B (D)
A8JC15	*pyr+*	0.5	0	chlo	chlo	0	no functional annotation	hydrolyzing O-glycosyl compounds; carbohydrate metabolic process; (not found)	no high confidence hits (<32%)	F (U)
A8JCS8	*pyr+*	0.5	0	chlo (thyl)	chlo	0	ACT domain (metabolism); amino acid binding	amino acid binding; metabolic process; chloroplast stroma	glycine cleavage system transcriptional repressor (100%; 59%)	-

UniProt ID	Up in *pyr+*/ *pyr-*	*pyr+* S.C.	*pyr-* S.C.	WoLF PSORT Local.	Pred Algo Local.	TMD	Phytozome function (PANTHER; KEGGORTH/KEG/GO)	Argot2 (molecular function; biological process; cellular component)	Phyre[2] structure (%confidence; %coverage)	CO_2 DE
A8JD42	*pyr+*	0.8	0	chlo (thyl)	chlo	0	domain of unknown function 1350 (in cyanobacteria and plants, may be involved in photosynthesis)	hydrolase; (metabolic process); chloroplast	esterase (98%; 52%), giberellin receptor (98%; 59%), hydrolase	-
A8JEA7	*pyr+*	4.5	1	nucl	other	0	regulator of chromatin condensation	DNA binding/metal ion binding; (negative regulation of GTPase activity); nucleolus	regulator of chromosome condensation (100%; 82%)	-
A8JEP0	*pyr-*	0	1	*chlo*	*other*	0	no functional annotation	(not found); (regulation of transcription, DNA-templated); (nucleus)	no high confidence hits (<18%)	-
A8JEQ7	*pyr+*	0.8	0	chlo	chlo	0	rhodanese-like domain	(tranferase activity); (not found); chloroplast envelope	thiosulfate sulfurtransferase (100%; 87%)	B (D)
A8JFF0	*pyr+*	0.7	0	chlo	chlo	2	no functional annotation	(not found); (not found); (integral component of membrane)	no high confidence hits (<21%)	-
Q84X71	*pyr-*	0.8	4	*mito*	*other*	0	ankyrin repeat and protein kinase domain-containing protein	(not found); (not found); plasma membrane	burrh DNA-binding protein (100%; 95%), ankyrin repeat	-

Supplementary Table S2. Comparison of proteins identified in *pyr+* versus *pyr-* (LC-MS/MS) with genes differentially expressed in wild-type and *cia5* mutant strains in response to low CO_2 (Brueggeman et al., 2012; Fang et al., 2012). The number of proteins identified in two or more datasets is listed for 158 differentially expressed (Total DE, including proteins up in WT or up in *pyr-*) or non-differentially expressed (non-DE) proteins. Differential expression of gene transcripts was significant if q-value<0.05 for either CO_2 concentration (C-effect) or for the *cia5* mutant compared to the wild-type (S-effect). Genes were either upregulated (positive C-effect) or downregulated (negative C-effect) in response to low CO_2.

	**Total DE**	Up in *pyr+*	Up in *pyr-*	**Non-DE**
**Bruegg. total DE**	64	50	14	73
C-effect, positive	12	10	2	9
C-effect, negative	52	40	12	64
**Fang total DE**	39	32	6	40
C-effect, positive	13	11	2	16
C-effect, negative	17	15	2	17
S-effect	31	24	4	32
**Bruegg. and Fang C-effect**	23	21	2	23
C-effect, positive	9	9	0	5
C-effect, negative	14	12	2	16

Supplementary Figure S1. Transmission electron micrograph of CAH3-deletion mutant (*cia3*, CC-2699). CAH3-deletion mutants retain the capacity to assemble a pyrenoid, both under high-CO_2 (left) and under low-CO_2 (right) growth conditions, and the matrix is traversed by tubules. Scale bars = 1 µm.

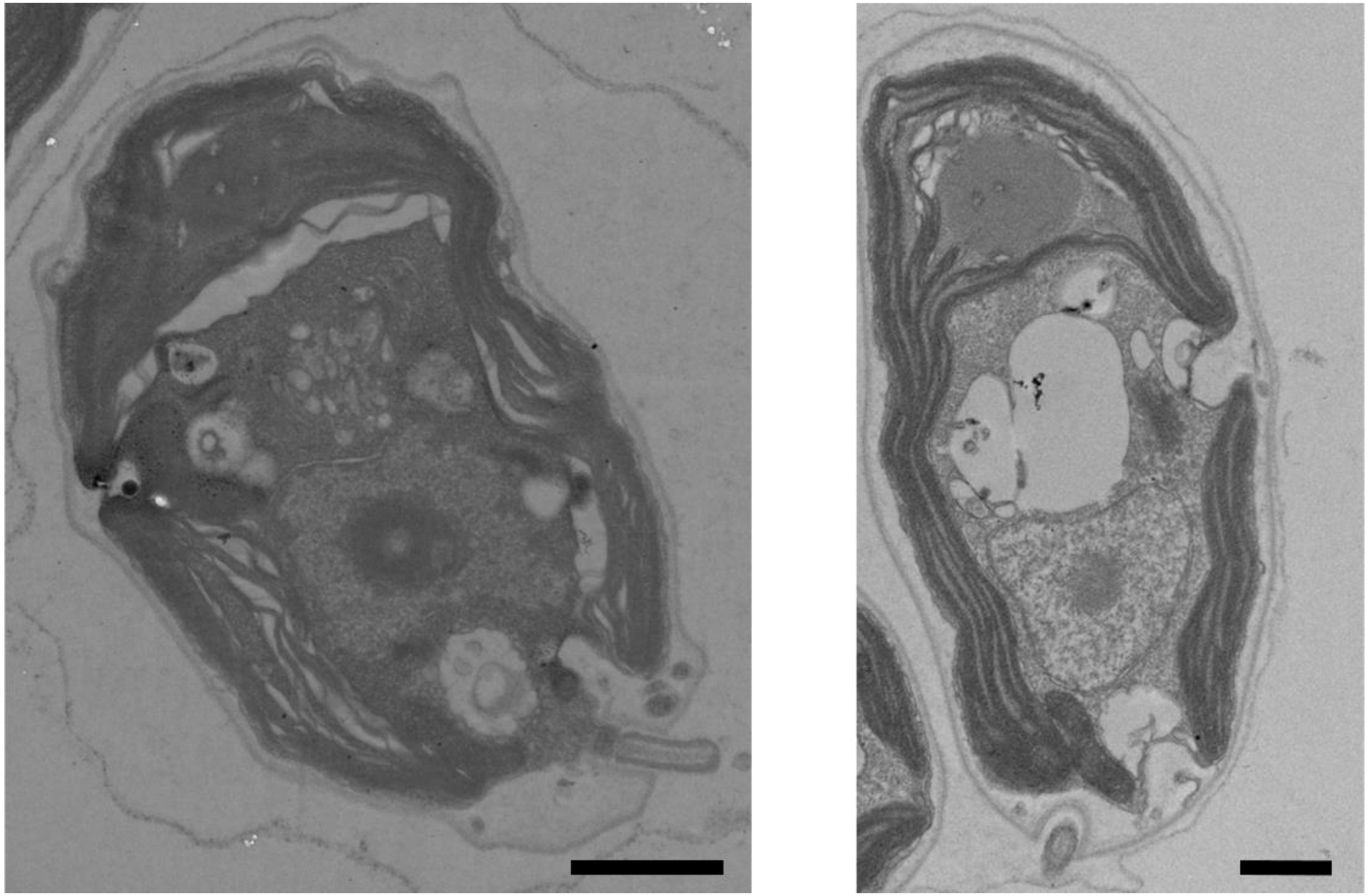

Additional references

Karlsson J, Clarke AK, Chen ZY, Hugghins SY, Park YI, Husic HD, Moroney JV, Samuelsson G. 1998. A novel α-type carbonic anhydrase associated with the thylakoid membrane in *Chlamydomonas reinhardtii* is required for growth at ambient CO_2. *EMBO Journal* **17**, 1208-1216.
